# Progress in Surgery

**Published:** 1896-11-28

**Authors:** 


### PROGRESS IN SURGERY.

### BRAIN TUMOURS.

It must be admitted that the success of operations for the removal of cerebral tumours has been somewhat limited. It is also true that surgical advance has failed to keep pace with the progress which has within recent years been made in the diagnosis and localisation of these conditions. Chipault states that of 123 cases of brain tumour operated upon 51 per cent. died from the effects of the operation. This is not to be wondered at when we bear in mind the deep-seated position and physical characters of so many cerebral neoplasms, which render attempts at removal more than dangerous. Before we can expect better surgical statistics we must gain still more diagnostic skill, both as to the position and the nature of the tumours, in order that the cases subjected to operation may be more carefully selected.

As illustrating the difficulties in diagnosis, Shaw draws attention to the very variable results which different pathological lesions produce in clinical cases (*Medical Press and Circular*, July, 1896). He states his opinion that the chart of the human encephalon, so far as its functions are concerned, will have to be corrected and completed by human pathological anatomy. He describes the clinical and pathological features of eighteen cases of his own, observed in a lunatic asylum, and from a study of them comes to the following conclusions: (1) That lesions in the occipital, temporal, supramarginal, precuneate, and prefrontal regions or cerebellum give rise to symptoms of general physical and marked mental weakness, with slight paresis or transient paralysis; while (2) permanent paralysis, with or without contracture or atrophy, is the outward expression of disease in the Rolandic area and parts immediately behind and below it, and of the central ganglia and internal capsule. (3) Where the central ganglia were the principal seats of lesions there was much less mental deterioration than in cases with extensively diseased cortex. (4) He found the extent and degree of paralysis in his cases bore very little proportion to the pathological lesion, and in many cases seemed to contradict the usual views on localisation. (5) In five of his cases in which the pupil on the paralysed side was the smaller there was ramollissement of the opposite cortex or cerebral medulla. Hæmorrhagic effusion over one hemisphere was accompanied by smaller size of the pupil on the opposite side. (6) Late pseudo-bulbar symptoms were probably caused by softening of the lenticular nucleus. (7) Exaggerated knee reflex and ankle clonus, as a rule, indicated cortical or ganglionic destruction; the absence of these reflexes was attributable to pressure from such causes as tumour, hæmorrhage, &c. (8) Tremor and twitching were usually the expression of disease of the lenticular nucleus, or of pressure on the cerebral medulla or cortex, or of lesions of the cerebellum. (9) Cutaneous anæsthesia was associated with disease of the limbic or falciform lobe, the caudate nucleus, the posterior limb of the internal capsule, or the Rolandic area. (10) Auditory symptoms were due to disease in the temporal lobe (not necessarily the posterior end of the first temporal gyrus). (11) Visual symptoms occurred with extensive lesions anywhere behind the fissure of Rolando. (12) Impairment of muscular sense was not pathognomonic of cortical lesions, as it occurred in sub-cortical disease.

In a paper by Stieglitz (*Amer. Jour. Med. Sc.*, May, 1896) the case is fully reported of a woman, aged 25, in whom a glio-sarcoma, the size of an almond, developed two years and ten months after the successful removal of a cystic tumour of the left motor area. It was excised, and recovery followed. In commenting on the case the writer comes to the following conclusions: (1) A new growth may exist without causing any general cerebral symptoms. The symptoms actually present are of far more diagnostic value than the absence of other customary symptoms. (2) A cystic tumour of the brain, not of parasitic origin, is not so innocent in character as some would suppose. (3) Simple evacuation of a cyst is not sufficient; if surgically possible, the sac should be removed. (4) It may even be justifiable to perform a second operation for removal of the sac if it cannot be done at the first. Another case is also reported of a man, 33 years of age, from whom a gliosarcoma was removed from the right cerebellar hemisphere. The patient died. The value of electric tests applied to the facial and auditory nerves was illustrated by this case.

The variations in symptomatology are further illustrated by a third case reported in the same paper. A spindle-celled sarcoma of the left arm, face, and speech centre was localised and removed, but with a fatal issue. Among the points of interest in this case may be cited: (1) That although a very large tumour was present in the brain, optic neuritis was absent. (2) A previous history of syphilis does not preclude the presence of a non-specific growth. In this and the previous case such a history was forthcoming, yet sarcomatous tumours were found. This raises the question how long antisyphilitic treatment is to be persisted in under such circumstances. Horsley says six weeks; Starr three months; the writer thinks much shorter periods suffice, three weeks of thorough treatment being long enough. (3) The absence of headache for five or six months, and of nausea, vomiting, and vertigo throughout is another point of interest. (4) The inefficiency of probing for a tumour with an aspirating needle was conclusively shown in this case.

A remarkable case reported by R. C. Bartlett (*Australian Med. Mag.*, February, 1896) is also of value as showing a diagnostic difficulty. A patch of dense sclerosis in the motor area gave rise to symptoms so like those of cerebral tumour, that an operation was advised and performed. The patient died 23 hours later. On post-mortem examination it was found that the area of sclerosis in the brain corresponded to a specially thickened part of the skull. It involved chiefly the superior frontal and adjacent parts of the middle and ascending frontal regions. A large, partially decolorised blood clot was found in the right lateral ventricle. Otherwise the rest of the brain was normal. The diagnosis of sclerosis of brain substance was confirmed by microscopic examination.

Passow met with a case in which the patient had suffered from middle ear disease for sixteen years, and consulted him for symptoms of cerebral compression. An operation was about to be performed for the clearing out of the mastoid process, but the patient died under chloroform. On post-mortem examination a glio-sarcoma of the corpora-quadrigemina was discovered (*Deutsch. Med. Wochensc.*, No. 44, 1895).

Two interesting cases are reported by Munro, in which recovery took place without surgical intervention, from symptoms strongly suggestive of cerebral tumour. In the first a boy of seven suffered from headache, vomiting, bilateral acute optic neuritis passing into consecutive atrophy, with persistent bilateral absence of knee-jerk, and slight paresis of left oculo motor nerve. He was treated by a simple mixture containing calomel and bicarbonate of soda, and most of his symptoms gradually passed off. The writer believes that the patient suffered from a cerebellar tumour, most likely tuberculous in nature.

The other case, a man, aged sixty-three, had a history of having suffered from a severe illness when sixteen years of age. It was characterised by severe headache and symptoms almost, if not absolutely, characteristic of intracranial tumour. He became blind during this illness. Recovery took place, and, except for an attack of typhus fever when twenty-nine, he enjoyed good health for about forty-five years. Death was due to cancer of the pylorus. On examining the brain postmortem a myxoma was found in the cerebellum. The writer concludes that his early illness was due to the myxoma, which became quiescent, and left no permanent ill-effect save the blindness (*Glas. Med. Journ.*, September, 1896).

Broca reports a successful operation for a cerebral tumour situated at the end of the fissure of Sylvius (*Med. Week*, May, 1896); and Pitt and Lane a case in which temporary alleviation of symptoms has followed the exploration of the skull in a case of sarcoma perforating the bone (*B. M. J.*, April, 1896).

---

### MIDDLE EAR DISEASE AND ITS SEQUELÆ.

**Acute Suppuration.**—A high opinion of the value of paracentesis of the tympanic cavity in cases of acute middle-ear suppuration is expressed by Weil. He believes that early tapping is a valuable means of avoiding many of the serious consequences, such as chronic otorrhœa, deafness, tinnitus, &c., which so often follow acute attacks. He also favours washing out with saline solutions or boracic acid, without, however, condemning the dry method of treatment. (*Centralbt. f. Chirurg.*, No. 20, 1896.)

Bruch describes a new canula with which he taps the middle ear in cases where a small perforation is situated high up in the region of Schrapnell's membrane. It is of metal, with a very small calibre, and is connected with a small injection syringe, the opening of which is placed laterally, so as not to interfere with the operator's view of the membrane while using it. He has not observed any trouble from giddiness while using this apparatus. (*Deutsch. Med. Woch.*, No. 4, 1896.)

**Cerebral Abscess.**—In a full and instructive paper in the *American Journal of Medical Science* for August, 1896, Eskridge discusses the diagnosis of chronic abscess of the brain. He divides chronic abscess into two classes, basing the sub-division on the mode of onset of the condition. In one class, after an acute fæcal intracranial suppuration, the symptoms have become latent for a time; and, in the other, the onset has been so ill-defined, and the progress so insidious, that the condition first manifests itself only when symptoms of intracranial tension are already well marked. The former abscess he calls latent, and the latter chronic, irrespective of the actual length of time each has existed. He emphasises the importance of the fact that all abscesses of the brain do not begin acutely, and of bearing in mind the wide clinical distinctions between an acute and a chronic cerebral abscess. The symptoms presented by a brain abscess being common to a variety of cerebral conditions, it is important in all cases carefully to investigate the mode of onset of the symptoms, and to inquire as to all possible causes of intracranial suppuration, in such directions, for example, as an antecedent injury to the head, inflammatory conditions in the ear, with or without suppuration, collections of pus in any part of the body, or the presence of such infective diseases as influenza, diphtheria, typhoid fever, &c. In the majority of cases of chronic cerebral abscess a history, more or less clear, of headache, irregular febrile attacks, dimness of vision, vertigo, and interference with the motor functions will be obtainable.

Of the individual symptoms, headache is the most constant and characteristic. It is variable as to duration, severity, and nature, but is almost always present, and in the terminal period of the disease, when œdema and softening ensues, it becomes very severe. The seat of the pain does not always correspond to the abscess. The temperature is usually normal or subnormal, sometimes with intermittent rises. Optic neuritis is more frequently present than absent, but is less marked than in cerebral tumour. The duration and nature of the terminal period depends on the manner in which the abscess causes death. This may take place (1) through the abscess bursting into the lateral ventricle; (2) through bursting into the surface of the hemispheres, causing lepto-meningitis, local or more likely diffuse; (3) bursting into the posterior fossa of the skull, and interfering with the parts at the base, so arresting respiration and heart action, or setting up cerebro-spinal meningitis; (4) œdema or softening of the brain, with gradual loss of its function, sometimes precedes the fatal issue. When the terminal stage has once set in it is usually progressive.

The localising of cerebral abscess is exceedingly difficult, partly because the commonest seats of such collections, *e.g.*, the tempero-sphenoidal, frontal, or cerebellar regions, are just those whose functions are ill-defined and little known. In the frontal lobe, for example, a large abscess may exist, and unless it extends sufficiently far forward to encroach on the motor areas, may be quite unsuspected. The condition of the pupil is of value as a guide; if sluggish and abnormal in size on the side opposite to that of muscular paralysis, the posterior part of the frontal lobe, or anterior part of the tempero-sphenoidal lobe, is indicated as the seat of the lesion. A successful case of operation for abscess in the frontal lobe is reported by Olmer (*N. York Med. Rec.*, May, 1896). It followed an inflammatory condition of the upper eyelid. There was choked disc of the left eye, some right-sided motor paresis, with normal deep reflexes, sub-normal temperature, and hard, irregular pulse. Three ounces of pus were evacuated.

Tempero-sphenoidal abscesses often give few or no localising signs, the history and general symptoms being all there is to go upon in diagnosis. If of large size it may involve the lower motor areas, or Broca's convolution, and so aid in its localisation. According to Macewen, paralysis of the third nerve on the side opposite to that of face and arm paralysis is suggestive of abscess in the anterior part of the tempero-sphenoidal lobe on the same side as the affected third nerve. Much help may be obtained by carefully observing the order in which different groups of muscles become paralysed. Poulsen found, out of seventeen cases of cerebral abscess, twelve in the tempero-sphenoidal lobe, four in the cerebellum, and one simultaneously in the cerebellum and occipital lobe (*Münch. Med. Wochen.*, No. 24, 1896). Zaufal and Pick report a case of abscess in the left temporal lobe in which optic aphasia was a prominent symptom. After evacuation by trephining the patient recovered (*Prager Med. Wochen.*, 1896, No. 5). When the abscess affects the occipital lobe the chief symptoms are paresis and muscular rigidity, hemianæsthesia, and hemianopia of the opposite side. The fibres involved are usually those of the posterior half of the internal capsule. The occipital lobe is the commonest seat of the so-called "idiopathic" chronic abscess (*i.e.*, due to suppuration at a distant part of the body).

When the anterior part of the cerebellum is the seat of abscess formation, cerebellar ataxia, with respiratory and cardiac difficulty, occur. Symptoms are few when the posterior part is involved. Heiman had a case of middle ear suppuration which developed signs of intracranial mischief. The skull and mastoid were opened, but no abscess found. He died soon after of rapidly progressing lung tubercle. On post-mortem examination the head symptoms were explained by the finding of an abscess in the right half of the cerebellum. (*Centralbl. f. Chirurg.*, No. 24, 1896.) Haydon also reports a case of recovery from an intracranial abscess, which he believes to have been cerebellar. (*Brit. Med. Jour.*, March, 1896.)

**Sinus Thrombosis.**—The case of a boy fifteen years old is reported by Poulsen. He had suppuration in his mastoid process following middle ear disease of some years' standing. Following an operation for the evacuation of this pus, swelling and tenderness appeared along the line of the internal jugular vein. The transverse sinus was exposed and an epidural abscess evacuated. The sinus contained a purulent thrombus, and, on incising it, a profuse hæmorrhage followed and necessitated plugging and abandonment of the operation. Recovery took place. (*Centralbl. f. Chirurg.*, No. 20, 1896.)

**Affections of the Labyrinth.**—Rueda cites the case of a boy three and a half years old who suffered from middle ear disease, followed by necrosis involving the under part of the vestibule, the posterior half of the external semicircular canal, the outer opening of the posterior semicircular canal, and the beginning of the first turn of the cochlea. Facial paralysis and loss of co-ordination, with inclination to fall to the affected side ensued, but, on the removal of the sequestrum, disappeared, and some degree of hearing was restored. (*Revue de Laryngol.*, No. 11, 1896.) Hanel reports a case where the two fenestræ on the inner wall of the tympanum were perforated by tuberculous granulations in a case of diffuse miliary tuberculosis in a three months' old child. (*Zeitschrift. f. Ohren Welkunde*, 1895.)

**Radical Operations for Chronic Suppurative Otitis.**—Cases of chronic suppurative otitis are divided for purposes of treatment into five groups by Jones. (*Brit. Med. Jour.*, May, 1896.) Group A includes (1) cases of simple inflammation of the tympanic cavity with perforation of the membrane; (2) inflammation of the tympanum accompanied by polypi unconnected with bone disease; (3) cases in which inflammatory granulations or polypi are attended by patches of roughened bone. These are amenable to treatment by washing, insufflation, and curetting. Group B includes cases of attic suppuration with or without caries of the ossicles, or where adhesions between the ossicles and tympanic walls have interfered with drainage and caused deafness, tinnitus and giddiness. In such the ossicles should be removed. In group C are placed cases of chronic suppuration in mastoid antrum or tympanic walls inaccessible by the external ear. Here the Stacke-Schwartze operation is the appropriate treatment. The cases in group D of caries and necrosis of the petrous bone, involving the destruction of important structures, or risk to life, necessitate the making of a permanent opening in the mastoid to ensure prolonged and thorough drainage. The cases in group E are rare—namely, cases of suppuration with hyperostosis or exostosis coexisting. On account of the extreme hardness of the bone the dental burr is the safest instrument to employ. Jones is of opinion that, except in tuberculous cases, cholesteatomata, and extensive caries, it is an advantage to leave some part of the mucous membrane of the cavities, rather than to have them lined by skin, which is apt to be troublesome from eczema, collections of epitheleal scales, and cutaneous secretions.

Mann advocates the employment of Mangoldt's method of epithelium sowing in the covering over of the cavity of the middle ear, antrum, facial canal, and other surface wounds following the radical operation. After thoroughly purifying the bone cavities and chiselling them flat, epithelium scraped from the healthy skin is spread over the surface, and a protective dressing applied. In about three weeks a large cavity can be covered over.

**Meningitis in its Surgical Aspects.**—In a lecture on this subject D'Arcy Power emphasises the error of dividing all cases of meningitis into "tuberculous" and "non-tuberculous." He prefers to classify them as "infective" and "non-infective." The infective forms are tuberculous, pneumonic, influenzal, suppurative, and, in rare case, typhoidal and malarial. In the non-infective group are placed congenital, traumatic, reflex, and hysterical cases. The presence of optic neuritis, retraction of the head, and tumid abdomen are valuable diagnostic symptoms, but their absence is not to be taken to exclude meningitis.

The diagnosis is always difficult, as a similar train of symptoms may occur in such widely different conditions as typhoid fever, pneumonia, influenza, the onset of exanthemata, &c. It is only by a careful analysis of the history, symptoms, and progress that a correct opinion can be arrived at, Tapping the sub-arachnoid space in the lumbar region, the most recent advance in the differential diagnosis of the various forms of meningitis is described at length. (See recent numbers of THE HOSPITAL.) The prognosis is exceedingly grave in the tuberculous variety; less so in the other forms. Little advance has been made in recent years in treatment. Lumbar puncture and trephining for the relief of tension have been useful in many cases, more especially when the cause was other than the tubercle bacillus. (*Clin. Jour.*, May, 1896.)

